# Cellular loci involved in the development of brain arteriovenous malformations

**DOI:** 10.3389/fnhum.2022.968369

**Published:** 2022-09-21

**Authors:** Zahra Shabani, Joana Schuerger, Hua Su

**Affiliations:** ^1^Center for Cerebrovascular Research, University of California, San Francisco, San Francisco, CA, United States; ^2^Department of Anesthesia and Perioperative Care, University of California, San Francisco, San Francisco, CA, United States

**Keywords:** brain arteriovenous malformations, endothelial cells, pericytes, smooth muscle cells, astrocyte, inflammatory cells

## Abstract

Brain arteriovenous malformations (bAVMs) are abnormal vessels that are prone to rupture, causing life-threatening intracranial bleeding. The mechanism of bAVM formation is poorly understood. Nevertheless, animal studies revealed that gene mutation in endothelial cells (ECs) and angiogenic stimulation are necessary for bAVM initiation. Evidence collected through analyzing bAVM specimens of human and mouse models indicate that cells other than ECs also are involved in bAVM pathogenesis. Both human and mouse bAVMs vessels showed lower mural cell-coverage, suggesting a role of pericytes and vascular smooth muscle cells (vSMCs) in bAVM pathogenesis. Perivascular astrocytes also are important in maintaining cerebral vascular function and take part in bAVM development. Furthermore, higher inflammatory cytokines in bAVM tissue and blood demonstrate the contribution of inflammatory cells in bAVM progression, and rupture. The goal of this paper is to provide our current understanding of the roles of different cellular loci in bAVM pathogenesis.

## Introduction

An abnormal mass of blood vessels named “nidus” is a main characteristic of brain arteriovenous malformations (bAVM), leading to the direct shunting of blood from the arteries to veins. There is no intervening capillary bed in the nidus (Rodrigues de Oliveira et al., 2020). The Patients with bAVM are at risk of intracranial hemorrhage (ICH) (Kim et al., 2011). Overall, bAVMs account for 25% of hemorrhagic strokes in adults < 50 years of age (Cordonnier et al., 2010), and up to 40% of bAVM patients die or remain functionally impaired within one-year after ICH (van Beijnum et al., 2009). The treatment of unruptured lesions has become controversial because the natural history of these patients may be less morbid than invasive therapies (Stapf et al., 2006; Mohr et al., 2010, 2012, 2014; Cockroft et al., 2012; Derdeyn et al., 2017). However, the mechanism of bAVM development is not fully understood and there is no specific medical therapy available for bAVM patients.

Mouse model studies identified several key factors that are crucial for bAVM initiation and progression (Walker et al., 2011a; Choi et al., 2012, 2014; Chen et al., 2013a, 2014a,b; Zhang et al., 2016a). Angiogenesis and AVM causative gene mutation in endothelial cells (ECs) are necessary for AVM development. Arterial and venous specification of ECs is a crucial step for development of normal vascular bed, which is determined by genetic factors, although surrounding cells and hemodynamic forces may also contribute to vascular remodeling (Liu et al., 2020). Among those genes that are involved in EC arteriovenous specification, abnormal NOTCH signaling has been detected in human bAVMs and both gain or loss of function of Notch in mouse lead to bAVM formation (Murphy et al., 2009; ZhuGe et al., 2009; Li et al., 2014). Although ECs have been identified as the primary cellular locus for AVM initiation (Walker et al., 2011b; Choi et al., 2012; Garrido-Martin et al., 2014; Park H. et al., 2021; Shaligram et al., 2021), other cellular loci, such as pericytes and microglia/macrophages, have also been shown to play roles in bAVM pathogenesis (Chen et al., 2013a; Zhang et al., 2016a; Winkler et al., 2018; Krithika and Sumi, 2021; Scherschinski et al., 2022). Both human and mouse bAVM vessels have less mural cell coverage than normal vessels, which is associated with vessel leakage and hemorrhage (Chen et al., 2013a; Winkler et al., 2018). Inflammation may promote bAVM progression. An abnormally high numbers of inflammatory cells like macrophages, neutrophils, and T lymphocytes have been detected in human and mouse bAVMs, even in unruptured specimens (Guo et al., 2012, 2014). Both Cx3cr1^+^ microglia and Ccr2^+^ macrophages are present in bAVM lesions of an *Alk1* deficient mouse model indicating that both microglia and macrophages are involved in bAVM pathogenesis (Zhang et al., 2016a).

Astrocytes respond to multiple insults and diseases by a process called reactive astrogliosis or astrocytosis (Hamby and Sofroniew, 2010; Liddelow et al., 2017; Escartin et al., 2021). Abnormal astrocytes with increased expression of glial fibrillary acidic protein (GFAP) and vimentin have been observed in human sporadic bAVMs which is associated with deregulated retinoic acid signaling (Thomas et al., 2021). Astrocytes are essential cellular component of neurovascular unit which surround brain vascular ECs by their endfeet, resulting in generation of a penetrable membrane named the glial limitans and induction of capillary formation (Winkler et al., 2019).

To date, the roles of different cellular loci in bAVM initiation and progression have not been fully studied. In this review, we have summarized what we know based on studies conducted on animal models and surgical resected bAVM specimens. Understanding the roles of each cellular locus will help us to design targeted therapeutic strategies to treat bAVM or prevent bAVM hemorrhage.

## Endothelial cells

Endothelial cells form the lumen of the capillaries, arteries, and veins. Brain ECs form a control barrier between blood and brain parenchyma and influence the blood vessel formation, coagulation, fibrinolysis, as well as regulation of vascular tone and neuroinflammation process. Furthermore, ECs take part in the pathogenesis of bAVMs.

### Gene mutation in Endothelial cells is necessary for brain arteriovenous malformations initiation

Gene mutations in ECs are essential for bAVM initiation. Mutation of genes in transforming growth factor beta (TGF-β) family and RAS-MAPK pathway have been linked to bAVM development.

#### Familial AVM

**FIGURE 1:**
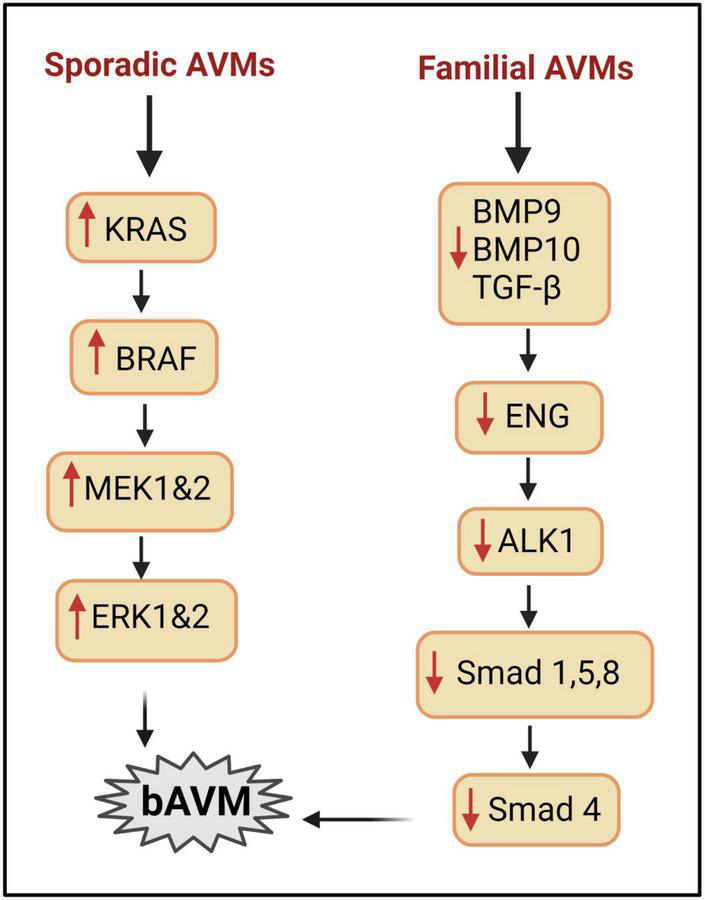
Signaling pathways involved in bAVM development. Right column: mechanisms implicated in HHT AVMs. BMP9/BMP10/TGFβ can regulate angiogenesis *via* interacting with ALK1/ENG to phosphorylate SMAD. Mutation of ALK1 or ENG reduces SMAD, leading to AVM development. Left column: mechanisms involved in sporadic bAVM. Mutations in genes of KRAS, BRAF pathway enhance the level of MEK and ERK and lead to bAVM development.

The majority of brain AVMs are sporadic; however, some evidence supports a familial component to the AVM phenotype, and that genetic variation is relevant to the disease course. Hereditary Hemorrhagic Telangiectasia (HHT) is an autosomal dominant disease. Majority of HHT patients have loss of function mutations of endoglin (*ENG*) or activin receptor like kinase 1 (*ALK1* also known as *ACVR1*) (Figure 1). ENG and ALK1 are receptors of TGF-β and bone morphogenetic proteins (BMPs), that are predominantly expressed in ECs. Some HHT patients have mutation in *SMAD4* or *BMP9*. TGF-β1 receptors, ALK 1 play an important role in the endothelial TGF-β signaling. Administration of low-dose TGF-β stimulates proliferation and migration of ECs through ALK1, whereas high doses of TGF-β result in quiescent endothelium (Lebrin et al., 2005).

Mouse studies showed that the lesional phenotypes are different between mice with heterozygous and homozygous *Alk1* or *Eng* mutation. In the Ad-Cre-treated brain of *Eng*^f/f^ mice, homozygous deletion of *Eng* is assumed because Eng-null ECs were detected. VEGF induced more severe vascular dysplasia in the Ad-Cre-treated *Eng*^f/f^ mice compared with the *Eng*^+/–^ mice (Choi et al., 2012). A robust and reproducible bAVM phenotype in adult mice were induced through brain focal angiogenic stimulation and *Eng* or *Alk1* conditional knockout (iKO) specifically in ECs (Chen et al., 2014b; Choi et al., 2014). Deletion of *Eng* or *Alk1* in pericytes or macrophages did not cause bAVM development (Chen et al., 2014b; Choi et al., 2014). Gene mutation in a small portion of ECs and in bone marrow derived ECs also are sufficient to cause bAVM formation (Walker et al., 2011b; Choi et al., 2012; Shaligram et al., 2021). Recently, Kim et al. (2020) demonstrated that overexpression of Alk1 can rescue the AVM phenotypes in *Alk1*- and *Eng* (iKO) mice *via* normalizing the expression of Notch and Smad target genes and restoring the effect of BMP9 on suppression of pAkt in *Eng*-deficient ECs. In normal physiological conditions, overexpression of Alk1 globally or in pan ECs does not cause vascular malformation (Kim et al., 2020). Therefore, reduction of endothelial Eng or Alk1 levels can lead to bAVM development in the presence of angiogenic stimulation.

Alk1 signaling regulates Notch ligands and its target genes, but Alk1-overexpression in normal ECs does not generate any changes in the expression of Notch targets. Notch signaling have been shown to play a critical role in normal vasculogenesis and angiogenesis, as well as in abnormal vascular remodeling. Increased expression of NOTCH-1 and downstream target HES-1 was observed in human bAVM tissue compared to control vessels (Murphy et al., 2009; ZhuGe et al., 2009; Li et al., 2014). Both activation and repression of Notch have been implicated in AVM development (Zhang et al., 2016b). Endothelial-specific activation of Notch-4 induced AVMs in mouse brain (Murphy et al., 2008). Like Notch-4, EC-specific, constitutively active Notch-1 results in vascular defects and AVM formation (Krebs et al., 2010). Deletion of recombination signal binding protein for immunoglobulin kappa J region (Rbpj), which block Notch signaling in ECs of postnatal mice, also caused bAVM phenotype (Nielsen et al., 2014). Decreased Notch signaling was found in *Alk1* knockout mouse models. These data suggest that there is a connection between the Alk1 and the Notch signaling during vascular morphogenesis (Larrivee et al., 2012).

A soluble form of ENG can be shed off from the ECs membrane affecting the TGF-β signaling required for angiogenesis by scavenging TGF-β ligands. Chen et al. (2009) showed that overexpression of soluble ENG caused bAVMs in mice. Soluble ENG also specifically binds to BMP9, leading to the inhibition of blood vessel formation (Castonguay et al., 2011) and participates in bAVM inflammation (Park E. S. et al., 2022).

BMPs also play an important role in ECs function and angiogenesis. Blocking BMP9 and BMP10 induced AVMs in the retina (Ruiz et al., 2016). Recent studies have indicated that BMP9 and BMP10 are probably the natural ligands for the ENG/ALK1 signaling pathway (Tillet and Bailly, 2015). BMP9 has high-affinity binding sites for both ENG and ALK1. When they bind ALK1, mRNA expression of ALK1 receptor signaling-dependent gene; transmembrane protein 100 (Tmem100) is induced within the arteries (Somekawa et al., 2012). Furthermore, disorganized arteries and downregulated Notch/Akt signaling were demonstrated in the Tmem100- deficient mice (Somekawa et al., 2012). It is thought that BMP9 and BMP10 promote the Notch pathway and thus suppress the arterial development and inhibit endothelial tube elongation (Roca and Adams, 2007). It appears that ENG competes with ACVR2B (type II receptor) for BMP9 binding site, and that ENG can be re-positioned by the type II receptor, possibly leading to activation of the type 1 receptor ALK1 and down-regulation of its targets like SMAD. Thus, it confirms the assumption that a deficiency of BMP9/10-ENG-ALK1-SMAD4 pathway is a possible mechanism of bAVMs development in HHT patients (Townson et al., 2012; Saito et al., 2017).

Moreover, recent studies have shown that neuropilin-1 (NRP-1) inhibits ALK1- and ALK5-mediated SMAD2/3 phosphorylation in ECs and modulates tip and stalk cell phenotypes in vascular sprouting and stretch-induced TGF-β1/ALK1 signaling in ECs when cocultured with SMCs (Korff et al., 2007; Aspalter et al., 2015). Neuropilin-1 (NRP-1) inhibits ALK1 signaling in tip cells during vascular sprouting (Aspalter et al., 2015). NRP-1 level is reduced in perivascular SMCs in the livers from patients with ALK1 mutation (Kilari et al., 2022). The mice with Nrp1 deletion in vSMCs and cardiomyocytes are viable, and present with decreased blood pressure, cardiac hypertrophy, and infiltration of perivascular inflammatory cells into the lungs (Wang et al., 2015). Another study reported that NRP-1 is involved in vSMC differentiation *via* platelet-derived growth factor (PDGF) signaling (Kofler and Simons, 2016). Nrp1 knockdown impairs PDGF-B driven vSMC migration (Pellet-Many et al., 2011). NRP-1 has a direct interaction with ENG and ALK1. NRP-1 deletion in vSMC leads to a decrease in ALK1/ENG signaling and to a decrease in pSMAD1/5/8 in vSMCs contributing to the formation of AVMs associated with HHT2 phenotype (Kilari et al., 2022). Although NRP-1 mutation has not be identified in HHT patient, it may involve in HHT pathogenesis through interaction with genes in TGF-β family and PDGF.

#### Sporadic AVMs

More than 95% bAVM cases are sporadic. Somatic mutations have been found in *KRAS/* MAPK pathway genes in the lesions of sporadic bAVM and peripheral AVMs (Couto et al., 2017; Al-Olabi et al., 2018; Nikolaev et al., 2018; Goss et al., 2019; Hong et al., 2019; Priemer et al., 2019). Expression of KRAS^G12V^ (a somatic mutation identified in sporadic bAVMs) in ECs *in vitro* stimulated ERK activity, and activated specific genes involved in angiogenesis and NOTCH signaling and enhanced EC migratory behavior. These effects of KRAS^G12V^ were reversed by inhibition of MAPK-ERK signaling using MEK inhibitor (Nikolaev et al., 2018). Further work is needed to understand the interplay between the MAPK-ERK pathway with VEGF and other angiogenic pathways.

Sporadic bAVMs models have been generated in mouse and zebrafish recently through somatic ECs-specific gain of function mutation in *Kras* (Fish et al., 2020). Fish et al. demonstrated that ECs-specific gain of function mutations in *Kras* (G12D or G12V) are sufficient to induce bAVMs in mice (Figure 1). Park et al. confirmed that *Kras* mutations promote bAVM development *via* the MEK/ERK pathway using a brain ECs –specific adeno-associated viral vector (AAVBR1) mediated brain ECs transfer of *Kras^G12D^* (Park E. S. et al., 2021).

### Endothelial inflammation

Endothelial cell inflammation in bAVM can be induced by multiple factors including hemodynamic changes as well as increased levels of angiogenic factors and cytokines in the lesion. Abnormally high flow rates and cerebral venous hypertension (VH) are common hemodynamic abnormalities in bAVM (Young et al., 1994). In rats, non-ischemic levels (15-23 mmHg) of VH cause expression of hypoxia-inducible factor 1 (HIF–1α) and its downstream signal, VEGF (Zhu et al., 2006). Further, HIF–1α, VEGF, SDF-1 expression, and neutrophils, macrophage, and MMP-9 activity are increased in the brain of the mice with VH. As shown in diabetic retinopathy, increased blood flow and vessel wall pressure in bAVM can cause EC damage and trigger EC inflammation (Stitt et al., 1995). ECs in bAVMs express cytokines and chemokines, which attract leukocyte infiltration causing vascular instability. The cellular adhesion molecules (CAMs), including E-selectin, intercellular CAM-1 (ICAM-1), and vascular CAM-1 (VCAM-1) are increased in ECs of human bAVMs and arteriovenous fistular in a rat model (Karunanyaka et al., 2008; Storer et al., 2008). Further receptors which are important in the inflammatory cascade like receptors for prostaglandin E2, a COX2-derived mediator of vascular remodeling, were found in the ECs and vSMCs and perivascular inflammatory cells (Keranen et al., 2021).

### Endothelial-to-mesenchymal transition

In bAVMs, endothelial-to-mesenchymal transition (EndMT) has been observed (Shoemaker et al., 2020; Li et al., 2021). EndMT is a process where mature ECs transform into mesenchymal cells by acquiring the characteristics of mesenchymal cells, characterized by invasiveness and proliferation, disorganization of ECs junctions and a spindle like morphology.

In cardiovascular, pulmonary, and hepatic developments, and some fibrotic diseases, such as renal, pulmonary, and hepatic fibrosis, TGF-β pathway plays a major role in regulating the EndMT (Perez et al., 2017). EndMT could not be defined by single protein. Shoemaker et al. found evidence of EndMT in bAVM through detection of the expression of EndMT-associated transcription factors (TFs) and mesenchymal markers including KLF4, SNAI1/2, VIM, ACTA2, and S100A4 (Pardali et al., 2017; Shoemaker et al., 2020). SMAD-dependent TGF-β signaling was not strongly activated in bAVMs and this pathway may be only partially involved in mediating bAVM EndMT. Other signaling pathways, such as Wnt/β-catenin (He et al., 2010), NOTCH (Nielsen et al., 2014), MAPK-ERK (Nikolaev et al., 2018; Li et al., 2021), and Sonic Hedgehog (Giarretta et al., 2021), may play roles. Upregulation of EndMT-associated genes was also reported in human umbilical cord ECs (HUVECs) over-expressing mutated KRAS (KRAS^G12V^), a somatic mutation that is associated with AVMs (Nikolaev et al., 2018).

In bAVMs that have microhemorrhage, immunohistochemical staining showed that the vascular endothelium exhibited decreased SMAD6 expression. Functional assays revealed that SMAD6 downregulation promoted the formation of ECs tubes with deficient cell-cell junctions (reduced the levels of VE-cadherin, occludin and ZO-1, and increased the level of N-Cadherin), and facilitated the acquisition of mesenchymal behavior (enhanced proliferation and migration) by ECs. Masson trichrome and immunofluorescence staining demonstrated that mesenchymal phenotype of ECs (increasing in the levels of the mesenchymal markers N-cadherin, α-SMA, SM22α, and CNN1 and reducing in the levels of the EC markers VE-cadherin, von Willebrand factor, Tie2, and CD31) is enhanced in bAVMs with microhemorrhage. TGF-β/BMP signaling mediated by SMAD6 in vascular ECs is associated with microhemorrhage in bAVMs. Therefore, mesenchymal behavior of ECs induced by SMAD6 downregulation is associated with bAVM microhemorrhage (Fu et al., 2020).

## Pericytes and vascular smooth muscle cells

**FIGURE 2:**
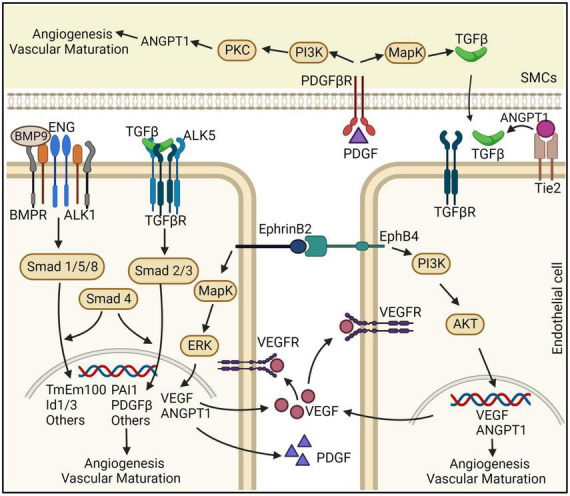
Schematic representation of the effects ECs and vSMCs on bAVM development. ENG and ALK1 are receptors of BMPs. TGF-β interacts with ALK5 and TGF-βR that are predominantly expressed in ECs. BMP9/10-ENG-ALK1 by triggering SMAD1,5,8, and 4 regulate angiogenesis and vascular maturation. Disruption of this pathway can cause the development of bAVMs. ANGPT/Tie2 are linked to TGF-β activation and binding to TGF-βR, which through SMAD2,3, and 4 induces angiogenesis. EphrinB2/EphrinB4 are differentially expressed in arterial and venous ECs. PDGFB and PDGFR-β both have an essential role in pericyte recruitment during angiogenesis. In the vSMCs, PDGFB upregulates ANGPT-1 through PI3K and PKC pathway and upregulates TGF-β1 expression through the MAPK pathway. ANGPT-1: angiopoietin 1, PDGFB: platelet derived growth factor B, PDGFR-β: PDGF receptor β, ECs: endothelial cells, SMCs: smooth muscle cells.

Pericytes and vSMCs are mural cells located at the abluminal side of small and large vessels, respectively (Armulik et al., 2011). Mural cells have a crucial role in vascular stability. Reduction of vascular pericytes impairs vascular integrity (Armulik et al., 2010; Bell et al., 2010). Pericytes modulate and maintain the BBB integrity through releasing signaling factors, such as angiopoietin 1 (ANGPT1), to determine the number of EC tight junctions and direct the polarization of astrocyte endfeet (Armulik et al., 2010). A reduction in pericyte numbers can cause a loss of tight junctions between EC, leading to increased BBB permeability (Winkler et al., 2013; Sengillo et al., 2013; Brown et al., 2019). In addition, PDGFB-PDGF receptor β (PDGFR-β), TGF-β, basement membrane and extracellular matrix (ECM) proteins, and pericyte differentiation have been shown to maintain normal brain vascular structure and function (Figure 2).

### Pericytes and vascular smooth muscle cells are reduced in brain arteriovenous malformations vessels

Mural cells including pericytes and vSMCs are considered as key components of the vascular wall and operate in maintaining healthy homeostasis. Pericytes cover almost 90% of brain vascular abluminal side. The vSMCs are placed in the tunica media. Together, they offer vessel stabilization and control blood flow *via* regulating dilation and contraction of vessels (Frosen and Joutel, 2018). The decreased mural cells in bAVM vessels makes them vulnerable to vascular leakage and subsequent microhemorrhage (Pan et al., 2021). Reduction of mural cell coverage in the bAVM vessels in mouse models and human specimens is associated with reduction of PDGFB and PDGFR-β protein levels in bAVM lesions and AVM bleeding (Walker et al., 2011b; Chen et al., 2013a; Winkler et al., 2018). Compared with normal brain angiogenic foci, the lesion in bAVM mouse models have more vessels with diameters larger than 15 μm that lack α-SMA positive cells. However, α-SMA positive cells in human bAVMs have yet to be fully characterized, and the functional consequences of other described abnormalities such as cytoskeleton and contractile proteins remain unclear (Wong et al., 2000; Uranishi et al., 2001; Kim et al., 2018).

### Pathways regulating mural cell coverage of brain arteriovenous malformations

Currently, the reasons of mural cells reduction in bAVM are unknown. Tie2/ANGPT-1 and PDGFB/PDGFR-β are two important pathways regulate mural cell-recruitment during vascular remodeling. We found that *ALK1* mutation decreases PDGFB expression in human and mouse brain ECs (Chen et al., 2013b). PDGFB upregulates ANGPT-1 in vSMCs through PI3K and PKC pathway and upregulates TGF-β1 expression through the MAPK pathway in vSMCs (Nishishita and Lin, 2004). In this section, we discussed three major pathways that regulate mural cell recruitment during angiogenesis and their involvement in AVM pathogenesis.

#### PDGFB and PDGFR-β

Both PDGFB and PDGFR-β have an essential role in pericyte recruitment during angiogenesis. PDGFB is secreted from the endothelium as a disulfide-linked homodimer and retained within the ECM as the result of electrostatic interactions (Abramsson et al., 2007; Andrae et al., 2008). This creates a steep perivascular concentration gradient of PDGFB which is essential for the recruitment of mural cells (Enge et al., 2002; Abramsson et al., 2007; Armulik et al., 2010). PDGFB upregulates ANGPT-1 in vSMCs through PI3K and PKC pathway and upregulates TGF-β1 expression through the MAPK pathway in vSMCs (Nishishita and Lin, 2004) (Figure 2).

Both pericytes and vSMCs express PDGFR-β (Tallquist et al., 2003). Global knockout *Pdgfb* or *Pdgfr*-β in mice results in the loss of pericytes from the microvessels (Lindahl et al., 1997) and cerebral hemorrhage (Hellstrom et al., 2001). Homozygous deletion of *Pdgfb* or *Pdgfr*-β in rodents results *in utero* death due to widespread hemorrhage (Hellstrom et al., 2001).

Abnormal expression of PGDFB and PDGFR-β has been described in bAVMs in rodent models and patients (Yildirim et al., 2010; Winkler et al., 2018; Zhu et al., 2018). Knockdown ALK1 in human brain microvascular ECs reduced PDGFB expression (Zhu et al., 2018). Pdgfr-β expression is reduced in the bAVM lesions of *Alk1*-deficient mice (Chen et al., 2013a).

Thalidomide and lenalidomide (one of the newer analogs of thalidomide) treatment improved mural cell-coverage of bAVM vessels and reduced bAVM hemorrhage (Zhu et al., 2018). Thalidomide restored Pdgfb expression in bAVMs. Overexpression of PDGFB in bAVM lesion mimicked the effects of thalidomide, suggesting that thalidomide reduces bAVM hemorrhage through upregulation of Pdgfb in bAVM.

#### ANGPT/TIE2

ANGPT is part of a family of vascular growth factors that are implicated in embryonic and postnatal angiogenesis. ANGPT is involved with controlling microvascular permeability, vasodilation, and vasoconstriction by signaling in vSMCs surrounding the vessels. There are four identified angiopoietins: ANGPT-1, ANGPT-2, ANGPT-3, and ANGPT-4 (Valenzuela et al., 1999). ANGPT/TIE2 signaling plays a role in the recruitment of peri-endothelial support structures, including pericytes and vSMCs (Figure 2). ANGPT-1, expressed by pericytes and vSMCs, is critical for vessel maturation, as well as cell adhesion, migration, and survival. ANGPT-2, expressed by ECs, promotes cell death, and disrupts vascularization. When it is in conjunction with VEGF, it can promote neo-vascularization (Hegen et al., 2004; Fagiani and Christofori, 2013).

Alternations of ANGPT/TIE2 expression have been found in human sporadic bAVM specimens (Hashimoto et al., 2001). ANGPT-2, which allows loosening of cell-to-cell contacts, is overexpressed in the perivascular region in bAVM vascular channels, while ANGPT-1 expression is not changed (Hashimoto et al., 2001). Therefore, imbalance of ANGPT/TIE2 signaling could be another cause of vessel wall defects in bAVM.

ANGPT-2 expression is also increased in HHT AVMs. *Alk1* germline deletion increase Angpt-2 expression in brain and spinal AVMs (Milton et al., 2012). PMP9/10 inhibition let to overexpression of Angpt-2 in the neonate retina (Ruiz et al., 2016). Angpt-2 expression was also increased in the postnatal retinal of *Smad4* mutant mice (Crist et al., 2019). Endothelial specific deletion of *Smad4* increased embryonic Angpt-2 expression (Lan et al., 2007). Administration of Angpt-2 monoclonal antibodies prevent and resolved retinal AVMs of *Smad4* mutant mice (Crist et al., 2019). Therefore, ANGPT/Tie2 may link to TGF-β pathway through SMAD4 (Crist et al., 2019).

However, some other studies showed that ANGPT-2 levels in the blood of HHT2 patients were decrease and unchanged in HHT1 patients (Ojeda-Fernandez et al., 2010). ANGPT-2 expression was also lower in outgrowth ECs of HHT1 and 2 patients than normal ECs (Fernandez-Lopez et al., 2007). This difference could be due to the changes of cultured cells and the different levels of ANGPT-2 in tissues and blood. In addition, ANGPT/Tie can signal through autocrine/paracrine (Takahara et al., 2004), their levels in the circulation may not influence their effects on AVM vessels. Taken together, Angpt/Tie2 pathway may play a key role in regulating mural cell plasticity. Dysregulation of this pathway contributes to the pathogenesis of bAVMs (Pan et al., 2021).

#### EphrinB2/EphB4

EphrinB2/EphB4 are differentially expressed in arterial and venous ECs, through angiogenic stimulation by VEGF and NOTCH (Adams and Alitalo, 2007). This signaling has been implicated in the regulation of vascular events, including sprouting angiogenesis, vascular morphogenesis, arteriovenous differentiation, and vascular homeostasis (Luxan et al., 2019). EphB4 is specifically expressed in ECs while EphrinB2 is expressed in ECs and their surrounding mesenchymal and mural cells including pericytes (Gerety and Anderson, 2002). EphrinB2 and EphB4 have been deemed as the primary molecular markers for endothelial arteriovenous specification and are responsible for the pericyte function and regulates the switch between normal and aberrant angiogenesis. It has been shown that diabetes increases the expression of Ephrin-B2 in the cerebrovasculature and pericytes. Concomitant increases in cerebral neovascularization parameters including vascular density, tortuosity and branching density in diabetic rats were accompanied by deterioration of cognitive function. Inhibition of Ephrin-B2 expression in pericytes significantly restored cerebral vascularization and improved cognitive functions (Coucha et al., 2019). EphrinB2/EphB4 is also a key regulator of intussusceptive angiogenesis (Figure 2; Groppa et al., 2018) through controlling the outcome of intussusceptive angiogenesis by fine-tuning the degree of ECs-proliferation caused by specific VEGF doses, without directly affecting VEGFR2 activity, but rather modulating its downstream signaling through MAPK/ERK. EphrinB2 is a crucial regulator of PDGFR-β expression in vSMCs, and thereby acts as a molecular switch regulating the downstream signaling activity induced by PDGFB/PDGFR-β.

Ephs are among few receptor tyrosine kinases known to attenuate MAPK signaling downstream of mitogens (Miao et al., 2001; Pasquale, 2008; Ottone et al., 2014). EphrinB2 suppresses VEGF- and ANGPT-1-induced RAS-MAPK activities (Kim et al., 2002). In skeletal muscles, inhibition of EphrinB2/EphB4 in the present of low level of VEGF increased vessel-diameters and pErk in ECs (Groppa et al., 2018).

Accumulating evidence suggest that EphrinB2/EphB4 signaling plays a crucial role in AVMs development and other cerebrovascular disorders (Bai et al., 2014). Embryos harboring homozygous mutations in *EphrineB2* and *EphB4* exhibited vascular defects and AVMs (Krebs et al., 2010). It was shown in an *in vitro* model of HHT2 that loss of *ALK1* gene blocked BMP9 signaling, resulting in reduced EphrinB2 expression, enhanced VEGFR2 expression and dysregulated ECs sprouting and anastomosis (Feghali et al., 2019).

## Astrocytes

Astrocytes are the most abundant cell type in the brain that are mainly involved in neuronal growth and survival, and reparation of nervous system by allowing the removal of dead neurons and pathogens (Rahman et al., 2021). Astrocytes have a key role in up taking and releasing the neurotransmitters, regulating ion homeostasis, and preserving the BBB integrity by secreting basement membrane proteins, such as laminin (Heithoff et al., 2021). Astrocytes also play roles in neuroinflammation (Colombo and Farina, 2016). Reactive astrocytes are astrocytes that go through morphological, molecular, and functional alterations around injured tissue following pathological conditions. Depending on context, reactive astrocytes may have fraction of simple changes between different states (Escartin et al., 2021).

### Role in normal brain function

Astrocyte is a cellular component of neurovascular unit. Brain vascular ECs are bordered by astrocyte endfeet, which build a penetrable membrane named the glial limitans, and have a role in the induction of capillary formation (Winkler et al., 2019). Astrocytes extend processes that physically link neighboring neurons with blood vessels (Hawkins and Davis, 2005; Vangilder et al., 2011), allowing them to sense changes in the neuronal microenvironment and adjust the microvasculature accordingly (Hawkins and Davis, 2005; Attwell et al., 2010; Gordon et al., 2011; He et al., 2012). Due to the anatomical and physiological contacts between astrocytes and ECs, any stimuli from pathological astrocytes can influence EC function (Zhou et al., 2019).

### Roles in brain arteriovenous malformations pathogenesis

**FIGURE 3:**
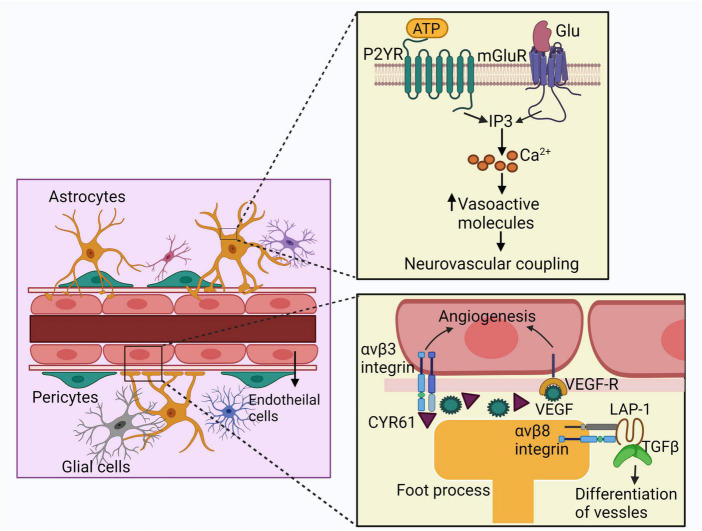
Schematic representation of the effects of astrocytes on bAVM pathogenesis. Astrocytes express mGluR and P2YR which interact with glutamate or ATP. Activation of these receptors trigger IP3 signaling cascade and the intracellular calcium currents, leading to the release of vasoactive molecules. These agents finally contribute to neurovascular coupling. Astrocytes also release VEGF in bAVM, which delivered to the ECs enhancing angiogenesis. Astrocytes also express CYR61, which takes part in angiogenesis. Foot processes of astrocytes express integrin αvβ8, interacting with LAP and activates TGF-β which promotes differentiation and maintenance of vessels. ECs: endothelial cells, mGluR: metabotropic glutamate receptors, P2YR: purinergic receptors, ATP: adenosine triphosphate, LAP: latency-associated peptide, IP3: inositol triphosphate.

The effects of astrocytes on bAVM pathogenesis are summarized in Figure 3. Several lines of evidence suggest that the astrocytes are involved in pathogeneses of bAVM. The expression of γ-glutamyl transpeptidase GGTP and glucose transporter 1 (GLUT1) protein expressed by ECs and astrocytes are increased in AVM nidus structures compared to control vessels confirming the contribution of astrocytes in bAVM pathogenesis (Thomas et al., 2018). Accumulation of albumin in astrocytes surrounding the AVM lesion has been noticed, which motivates the conversion of astrocytes from a resting state to a reactivate stage *via* triggering TGF-β signaling (Raabe et al., 2012). Li et al. reported that astrocytes in bAVM lesion have a higher level of VEGF and illustrated that the VEGF released by astrocytes can be delivered to the vascular ECs *via* perivascular pedunculus structures (Li et al., 2018). Patients with recurrent bAVM have an increased VEGF expression in astrocytes compared to non-recurrent bAVM cases, suggesting that astrocytes play roles in bAVM development (Zhou et al., 2019).

In a recent study, immunohistochemical analysis detected irregular astrocytes in and around bAVM nidus. The retinoic acid signaling pathway, which leads to the expression of angiogenic gene CYR61, was upregulated in the astrocytes in and around the bAVM structure. These data shows that astrocytes can modulate angiogenesis around the AVMs (Thomas et al., 2021). Notably, CYR61 gene takes part in angiogenesis through activating several growth factors, enhancing the migration and adhesion of ECs, as well as over-expression of αv integrin subunit and MMP, and other genes that are responsible for angiogenesis (Chen et al., 2001).

It has shown that the foot processes of perivascular astrocytes in adult mice and rats express integrin αvβ8, which interacts with latency-associated peptide (LAP) and activates TGF-β promoting differentiation and maintenance of vessels (Cambier et al., 2005). The level of integrin β8 protein in perivascular astrocytes in human bAVM lesion is lower compared to normal brain tissue (Su et al., 2010). In addition, elimination of integrin αv or β8 in astrocytes disrupts the suitable contacts between astrocyte endfeet and the vascular ECs (Proctor et al., 2005). As well, elimination of integrin β8 increased dysplastic vessels and hemorrhage in the *Alk1*^+/–^ mouse brain (Ma et al., 2016).

Astrocyte crosstalk with brain ECs and pericytes by releasing soluble factors, including cytokines (Banks et al., 2018). Vasoactive molecules like arachidonic acid (AA), prostaglandin E2 (PGE2) and K+ generated by astrocytes endfeet onto the vSMCs, can regulate vascular tone (Watkins et al., 2014).

In addition, astrocytes are important contributors in normal structural integrity of newly formed vessels. During vascular tube generation, tight junction development is strongly associated with the cellular interactions between ECs and pericytes, followed by astrocytes (Bonkowski et al., 2011). In this regard, the interaction between pericytes and ECs is partially regulated by ECM. Both ECs and pericytes secrete MMPs which are important not only for ECM remodeling, but also for tight junction cleavage (Thanabalasundaram et al., 2011). Additionally, the endfeets of astrocytes, in association with pericytes, preserve the expression of ECs tight junction proteins, transporters and enzymes (Watkins et al., 2014). Therefore, the crosstalk among brain ECs, pericytes, and astrocytes results in tight junction formation during vascular tube development, which finally increases vessel wall integrity.

The above-mentioned data confirms the crucial effects of perivascular astrocytes in the neurovascular coupling. Astrocyte malfunction can lead toprogression of bAVM through impairment of BBB structure as well as the function of ECs, pericytes and vSMCs by cross talking among these cells.

## Inflammatory cells

**FIGURE 4:**
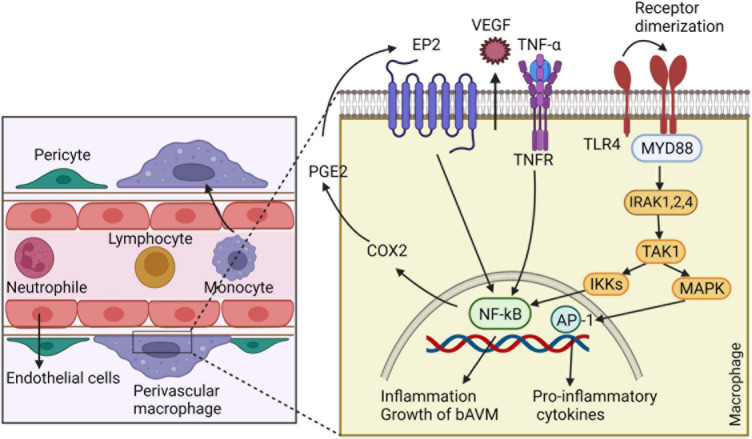
The involvement of inflammatory cells in bAVM growth. Macrophages release VEGF, which regulates the development of ECs and vessel sprouting. By generating matrix proteases, macrophages degrade ECM to create a guiding pathway for proliferation and migration of ECs and facilitating vascular branching. Macrophages also have crucial role in recruitment of pericytes around new blood vessels by expressing high levels of PDGFB. Macrophages through triggering autocrine feedback loop of COX2, PGE2, EP2-NF-kB-COX2 results in bAVM prograssion. Likewise, macrophages express TLR4, which binds to myeloid differentiation primary response gene 88 (MyD88) and leads to the generation of complex with IRAKs and activation of inhibitor kappa B kinase β (IKKβ). This process leads to translocation of NF-κB into the macrophage nucleus and overexpressing genes involved in inflammation. ECs: endothelial cells, COX2: cyclo-oxygenase 2, PGE2: Prostaglandin E2- EP2: prostaglandin E receptor subtype 2, MyD88: myeloid differentiation primary response gene 88, PDGFB: Platelet-derived growth factor B, TLR4: toll-like receptor 4, IRAKs: IL-1R-associated kinases, IKKβ: inhibitor kappa B kinase β.

Typically, host immune system is divided into innate and adaptive immunity. Innate immune cells contain macrophages, neutrophils, monocytes, plasmacytoid dendritic cells, natural killer (NK) cells, and other myeloid and lymphoid cells. Adaptive immune reaction is mediated by the immunoglobulin family and cells such as B- and T-lymphocytes (Netea et al., 2019). Present evidence shows higher inflammatory cytokines in bAVM tissues and blood, suggesting the contribution of inflammation in the pathogenesis, progression, and rupture of bAVM (Zhang et al., 2016a). The overall roles of inflammatory cells in bAVM pathogenesis are summarized in Figure 4.

### Microglia and macrophages

#### The normal function of microglia/macrophages

Microglia are ubiquitously distributed within the brain maintaining brain homeostasis. During disease or trauma, microglia may become activated, and the degree of microglia activation is directly correlated to the type and severity of brain injury (Speth et al., 2005). Activation of microglia is directly associated with dysfunction of the BBB by changing tight junction protein expression and increasing BBB permeability (Zlokovic, 2008). Macrophages are the primary BM-derived cells infiltrate into the brain angiogenic foci (Hao et al., 2008). These cells are primarily involved in phagocytosis and attracting peripheral immune cells to the injury site.

#### The microglia and macrophages are accumulated in and around brain arteriovenous malformations lesion

Macrophages are main inflammatory components that reside in and around vascular walls in human and animal bAVM samples with or without hemorrhage, indicating that macrophage burden is not a result of hemorrhage (Guo et al., 2014). A recent report shown that mononuclear cells (macrophages, T cells, B cells) were the dominant types of inflammatory cells in bAVMs (Wright et al., 2020). In addition, the macrophage migration inhibitory factor (MIF), a main activation factor of macrophages, is highly expressed in bAVM and participates in the vascular cell proliferation and apoptosis (Chen et al., 2012).

It must be noted that, although *Eng* deficient mice show impaired monocyte migration toward injured site (Zhu et al., 2017), the macrophage burden is increased in bAVM in both *Eng*- and *Alk1*-deleted animal models (Chen et al., 2013b; Choi et al., 2014). The persistent infiltration and pro-inflammatory differentiation of monocytes might cause increase of macrophage in bAVM (Zhang et al., 2016a). Accordingly, a study by Zhang et al. (2016a) confirmed the recruitment of fewer macrophages to the brain angiogenic area at the initial step after angiogenic stimulation and more macrophages burden in the later stage of bAVM development. Moreover, Eng-null macrophages showed slower but more persistent infiltration into the brain angiogenic regions compared to the normal macrophages; therefore, macrophages burden could be partially due to the continues infiltration and late clearance of BM-derived macrophages (Shen et al., 2014; Zhang et al., 2016a). Moreover, an *in vitro* system mimicking angiogenic niches using the co-culture of ECs and vSMCs illustrated that the HHT CD34^+^ monocytes have tendency to be differentiated into macrophages compared to normal CD34^+^ monocytes, indicating a pro-inflammatory feature of HHT monocytes (Zhang et al., 2016a).

Notably, a recent study revealed enhanced soluble ENG in human bAVMs. The soluble ENG motivates microglia to express angiogenic/inflammatory mediators like VEGF, TNF-α, IL-6, NLRP3, ASC, Caspase-1, and IL-1β, and proteolytic enzyme of MMP-2 and MMP-9, which mediate dysplastic vessel formation. This study indicates that microglia may contribute to soluble ENG-induced EC dysfunction *via* expressing inflammatory and angiogenic factors (Park E. S. et al., 2022). The microglia-mediated EC dysfunction can be a mechanism underlying soluble ENG-induced bAVMs observed in a previous study (Chen et al., 2009).

Another data suggested the high wall shear stress (WSS) as a critical parameter in aggregation of macrophages by activating proinflammatory signaling in ECs, mostly through activation of NF-κB, macrophage chemoattractant protein 1 (MCP1), and VCAM-1 (Ahn et al., 2016). As a result, macrophage aggregation in bAVM leads to uncontrolled inflammation, which increases abnormal vascular remodeling and worsens bAVM phenotype (Zhu et al., 2017). Therefore, addressing the mechanisms of macrophages action in vascular remodeling, bAVM pathogenesis and progression can improve therapeutic strategies alternate to surgery.

Macrophages enhance bAVM phenotype severity through stimulating angiogenesis, activating EP2-NF-KB-COX2 and TLR4/MyD88 pathways and secreting ECM-degrading proteases. During development and tissue healing or regeneration, macrophages stimulate angiogenesis, and facilitate tissue remodeling by secreting a number of proteases and growth factors (Krenkel and Tacke, 2017). The functions of macrophages in tissue and vessel repair include (i) secretion of pro-inflammatory cytokines and chemokines to maintain initial leukocytes infiltration, (ii) removal of invading pathogen and necrotic cell debris through phagocytosis, (iii) releasing MMP for ECM remodeling, (iv) promoting angiogenesis through guiding the sprouting of new blood vessels and stimulating the proliferation of EC and smooth muscle cells (SMC) (la Sala et al., 2012). The TIE2-expressing macrophage is a subtype of highly angiogenic macrophages that is able to influence angiogenesis *via* the ANGPT-TIE pathway (la Sala et al., 2012). It has been found recently that; microglia and macrophage promote angiogenesis by regulating EC subsets through SPP and IGF signaline pathways following spinal cord injury (Yao et al., 2022). Macrophages also have crucial role in recruitment of pericytes around new blood vessels by expressing high levels of PDGFB (Spiller et al., 2014).

Alternative to angiogenesis regulation, macrophages can act through an autocrine feedback loop of Cyclo-oxygenase 2 (COX2)- prostaglandin E2 (PGE2)- prostaglandin E receptor subtype 2 (EP2)-NF-kB-COX2 signaling pathway (Keranen et al., 2021). COX2, an inflammation-associated enzyme, is a main modulator of creation and progression of aneurysms (Keranen et al., 2021). In addition, the expression of COX2 in the vessels’ lumen or medial layer of human bAVMs is increased and exerts an important role in the growth and remodeling of the bAVM vessels. Further, COX2-derived PGE2, a key mediator of vascular remodeling, is enhanced in the vSMCs, ECs, and perivascular inflammatory cells of bAVMs (Keranen et al., 2021). On the other side, the expression of COX2 intensifies the inflammatory response through the loop of COX2-PGE2-EP2-NF-kB-COX2 signaling in macrophages (Keranen et al., 2021). In this context, a recent data illustrated a link between EP2 and COX-2 with macrophage burden in human intracranial aneurysm (Aoki et al., 2017). Administration of EP2 antagonist dramatically decreased macrophage infiltration and reduced the progression of intracranial aneurysm, suggesting the therapeutic potential of EP2 antagonists in vascular lesions (Aoki et al., 2017).

Likewise, macrophages are implicated in bAVM pathogenesis by acting through toll-like receptor 4 (TLR4), which is strongly expressed on macrophages (Mitsui et al., 2020). Binding of TLR4 to MyD88 leads to the generation of complex with IL-1R-associated kinases (IRAKs). This complex increases the formation of inflammatory cytokines, including IL-1β, IL-6, MCP-1, and C-X-C motif ligand (CXCL) (Michelsen et al., 2004). The secreted inflammatory cytokines and chemokines are implicated in the progress of aneurysmal rupture by compelling macrophages toward the pro-inflammatory phenotype (Mitsui et al., 2020). Totally, the signaling cascade *via* MyD88 activates inhibitor kappa B kinase β (IKKβ), which finally induces the phosphorylation and degradation of inhibitor kappa B (IκB). This process leads to translocation of NF-κB into the macrophage nucleus and overexpressing several genes involved in inflammation (Aoki et al., 2017).

Given the previous findings, macrophages have strong role in bAVM pathogenesis promoting progression in several ways, therefore, blocking the signaling pathways may promisingly be used for treatment of bAVM.

### Neutrophils

Neutrophils, the innate immune phagocytes, are critical effectors of the acute immune response against infection and tissue injury, with the capability to adjust their phenotype in accord with the microenvironment (Scalerandi et al., 2018).

Although the role of neutrophils in bAVM have been investigated in only a handful of studies so far, the presence of very large number of neutrophils in the vascular walls of bAVM indicate the involvement of these inflammatory cells in bAVM pathogenesis (Chen et al., 2008). Furthermore, the histological examination of surgically resected bAVMs tissue samples (*n* = 85) illustrated perivascular neutrophil recruitment and adhesion of neutrophils to the vessels’ walls of bAVM nidal in 60% (51/85) of the samples (Jarvelin et al., 2020). Neutrophil-lymphocyte ratio (NLR) is considered as an efficient way for assessment of the inflammatory action in the vessels. A recent study reported the significantly association between the higher NLR with poor outcome of cases with ruptured AVMs (Zhang et al., 2018). Neutrophils could promote bAVM progression through formation of neutrophil extracellular traps (Bonaventura et al., 2020; Shimada et al., 2021) and promoting angiogenesis (Massena et al., 2015; Wang J. et al., 2017). In addition, the complex of MMP-9/neutrophil gelatinase-associated lipocalin (NGAL) is increased in AVM samples. NGAL secreted by the neutrophils protects the MMP-9 from degradation and thus enhances the activity of MMP-9 (Ardi et al., 2007).

### Lymphocytes

It has been indicated that several kinds of inflammatory cells such as neutrophils, eosinophils, macrophages, and lymphocytes are present in bAVM samples (Wright et al., 2020). Of note, few studies focused on the involvement of lymphocytes in bAVM pathogenesis. Chen et al. (2008) showed that the bAVM specimens had minimal T-cells or B cells compared to macrophages and neutrophils. An another study (Guo et al., 2014) showed that the macrophages were dispersed frequently in the vessel walls and intervening stromal areas. T-lymphocytes were predominantly detected in unruptured bAVM tissue. However, rare B-lymphocytes and plasma cells were detected in the samples. They were generally appeared in samples with a large quantity of T-lymphocytes and were co-localized with the T-lymphocytes. Regardless the existence of lymphocytes in bAVM tissue, the effects of these cells on the bAVM development and progression are remained blurred and there is a need to be discovered.

## Discussion/summary

Much progress has been made in understanding bAVM pathogenesis. However, the effect of cellular population on development and progression of bAVM is still in its nascent stages. Although gene mutation in ECs seems essential for bAVM initiation, different cell types of the neurovascular unit and inflammatory cells are involved in bAVM pathogenesis. In this review, we systemically reviewed the cell types that present in bAVMs and their possible role in bAVM initiation, progression and hemorrhage.

The importance of EC in bAVM initiation has been supported by both animal study and analyzing human bAVM samples. Homozygous mutation of *Alk1* or *Eng* in a portion of somatic ECs (Walker et al., 2011b; Choi et al., 2012) is sufficient to trigger *de novo* bAVM in the presence of angiogenic stimulation in **adult** mice. Sporadic bAVM and extra-neural AVM harbor mutations of genes in RAS-MAPK pathways in a small number of ECs (Couto et al., 2017; Al-Olabi et al., 2018; Nikolaev et al., 2018). Transducing *Kras^G12D^ and Kras^G12V^* into brain ECs induced bAVM in mice (Fish et al., 2020; Park E. S. et al., 2021). Other abnormalities of ECs such as EC inflammation, and EndMT also contribute to bAVM progression and hemorrhage.

Although mutation of HHT causative genes in pericytes, vSMCs or macrophages did not trigger AVM initiation (Chen et al., 2014b; Choi et al., 2014; Garrido-Martin et al., 2014), abnormalities of these cells have been observed in bAVMs. Both human and mouse bAVM vessels have fewer mural cell coverage, which is associated with vessel leakage and hemorrhage (Chen et al., 2013b; Winkler et al., 2018), suggesting roles of pericytes and vSMCs in bAVM pathogenesis. In addition, pathways, including PDGFB/PDGFR-β, ANGPT/TIE2 and EPHRINB2/EPHB4 play roles in the reduction of the mural cell coverage on bAVM vessels.

An abnormally high number of inflammatory cells including macrophages, neutrophils, and T lymphocytes have been detected in human and mouse bAVMs, even in unruptured specimens (Guo et al., 2012, 2014). Abnormal astrocytes with increased expression of GFAP and vimentin have been observed in human sporadic bAVMs, which is associated with deregulated expression of genes in retinoic acid signaling, (Thomas et al., 2021). Their role in bAVMs pathogenesis also discussed in this review paper.

In summary, we have discussed roles of different cell-types present in bAVM in bAVM pathogenesis and possible underlying mechanisms. We hope the information provided in this review will help in identifying targets for developing new therapies for treating bAVM or preventing bAVM hemorrhage.

## Author contributions

ZS, JS, and HS drafted the manuscript. HS critically read the final manuscript. All authors contributed to the article and approved the submitted version.
